# Bonus? No, just an onus

**DOI:** 10.1590/S1516-31802012000400001

**Published:** 2012-09-11

**Authors:** José Otávio Costa Auler, Paulo Manuel Pêgo-Fernandes, Benoit Jacques Bibas

**Affiliations:** I PhD. Titular Professor, Discipline of Anesthesiology, and Director of Faculdade de Medicina da Universidade de São Paulo (FMUSP), São Paulo, Brazil.; II PhD. Associate Professor of the Discipline of Thoracic Surgery, Hospital das Clínicas (HC), Faculdade de Medicina da Universidade de São Paulo (FMUSP); Scientific Director of Associação Paulista de Medicina, São Paulo, Brazil.; III MD. Trainee in Tracheal Surgery and Respiratory Endoscopic Therapy, Discipline of Thoracic Surgery, Hospital das Clínicas (HC), Faculdade de Medicina da Universidade de São Paulo (FMUSP), São Paulo, Brazil. Faculdade de Medicina da Universidade de São Paulo (FMUSP), São Paulo, Brazil

On September 2, 2011, the Brazilian Ministry of Health and Ministry of Education published Interministerial Ordinance Nº. 2,087 in the Federal Official Gazette, which instituted the “Program for Placing Value on Primary Care Professionals”.^1^ This program comprises a set of measures to “stimulate” healthcare professionals (physicians, nurses and dentists) to work within the Family Health Strategy in localities with access and supply difficulties or with populations presenting greater vulnerability.^1^

For physicians, in addition to specialization, participation in the program will yield a bonus score of 20% in public competitions for medical residence in any specialty for those who remain in the program for two years (or 10% for one year).^2^ The program is expected to come into effect at the time of the public competitions for medical residence positions that will be available in 2013. The idea is to bring into the program 2000 physicians, 1000 nurses and 700 dentists, paid directly by municipalities. The municipalities that were to be included in the program would be defined by the end of 2011.^2^ The National Council for Medical Residence will publish the indices and criteria for scoring, through a Resolution.^2^

It is known that remoter areas of Brazil suffer from shortages of healthcare professionals.^2^^,^^3^ It has been estimated that around 1000 Brazilian municipalities currently do not have any physicians, which causes significant harm to their populations through becoming victims of lack of care.^3^ Attacking this problem was a measure agreed between the Ministries of Health and Education, and ought to be supported by the medical profession. However, this measure, which was implemented without proper dialogue with medical professional bodies or university medical schools, raises a series of issues that we intend to discuss here.

Recently graduated physicians, without medical residence and therefore without its training, do not yet have the qualifications, maturity and experience that are necessary in the profession, and they will end up exposing themselves and their patients to defective practicing of medicine.^4^ How can they practice family medicine if they have never had any training for this? Moreover, no information was released regarding where these physicians would be going, how they would work, what type of supervision they would have (if any), housing accommodation conditions and the method that would be used to determine where they would be going.

The current situation, in which new graduates coming out from medical courses have not been choosing to follow specialized training in family and community medicine, has been recognized.^3^ Even though the numbers of medical residence vacancies in this specialty are insufficient, a large proportion of them remain unfilled. If medical residence vacancies in family and community medicine are remaining unfilled (and also in intensive care, pediatrics, preventive and social medicine, gynecology and obstetrics etc.), it is clear that such bonuses will not be determining factors for individuals who wish to follow these fields. It will only be thus for individuals who want to enter the more highly sought programs. The high demand for these programs usually comes from market-based criteria that have little to do with social necessities.^3^ The big task to be undertaken would be to stimulate young physicians to study for these specialties and encourage them to leave the major centers and head for the interior of the country. For this, it would be necessary to create state career structures for physicians working in the national health system (Sistema Único de Saúde - SUS), with exclusive dedication, full-time employment, admission only by means of public competition, salary and career prospects compatible with their prolonged training and high professional responsibility.^3^^,^^4^

Because there are not enough medical residence vacancies for all newly graduating physicians, there is a high level of competition for residence places. The bonuses compromise the pillar that sustains universal public competitions: the merit of practical and theoretical knowledge, in this case acquired through the six years of the medical course.^4^ The bonus awards will cause distortion in the selections, thus reducing the meritocracy of access to the programs. Entry should take place according to the merit and capacity of each individual, with fair competition and without privilege or distinction between the candidates*.*

Thus, it seems that Brazil is going against the tide of history. While American universities seek to attract brilliant students to their folds,^5^^,^^6^ aiming to find future leaders in their fields, Brazil seeks ways of correcting basic deficiencies in its educational system through arbitrary measures that do not get to the root of the issue. Such measures only make a distorted correction of the truth.

Perhaps it would be more appropriate to call this program a “Program for DEVALUING primary care professionals”, given that at least in relation to those in the medical profession, they should ideally have gone through medical residence, i.e. good training in the field in which they are going to work, which is certainly not the case of newly graduated physicians. Professionals acquire value through good undergraduate medical training, appropriate training after graduation, in the field in which they will work, i.e. good MEDICAL RESIDENCE, and respectable working conditions and remuneration.

Do the ends really justify the means?
